# Sexual function, mental health, and quality of life under strain of COVID-19 pandemic in Iranian pregnant and lactating women: a comparative cross-sectional study

**DOI:** 10.1186/s12955-021-01720-0

**Published:** 2021-03-01

**Authors:** Negin Mirzaei, Shahideh Jahanian Sadatmahalleh, Mahnaz Bahri Khomami, Ashraf Moini, Anoshirvan Kazemnejad

**Affiliations:** 1 Department of Reproductive Health and Midwifery, Faculty of Medical Sciences, Tarbiat Modares University, Tehran, Iran; 2 Monash Centre for Health Research and Implementation, School of Public Health and Preventive Medicine, Monash University, Clayton, VIC Australia; 3 Breast Disease Research Center (BDRC), Tehran University of Medical Sciences, Tehran, Iran; 4 Tehran University of Medical Sciences, Tehran, Iran; 5 Department of Endocrinology and Female Infertility, Reproductive Biomedicine Research Center, Royan Institute for Reproductive Biomedicine, ACECR, Tehran, Iran; 6 Department of Biostatistics, Faculty of Medical Sciences, Tarbiat Modares University, Tehran, Iran

**Keywords:** COVID-19, Pregnant women, Lactating women, Sexual function, Quality of life

## Abstract

**Background:**

The impact of COVID-19 pandemic on mental health of pregnant and lactating women is unclear. This study aimed to assess the impact of COVID-19 on psychological health, sexual function, and quality of life (QoL) in Iranian pregnant and lactating women and compare the results with non-pregnant/non-lactating women.

**Method:**

This comparative cross-sectional study was carried out on pregnant and lactating women, with non-pregnant/non-lactating women from May to Jun 2020. Patients were asked to complete three questionnaires: Hospital Anxiety and Depression Scale (HADS), Female Sexual Function Index (FSFI), and Short-Form Health Survey (SF-12). One-way ANOVA was used to reveal the statistical differences between the three groups**.**

**Result:**

The mean age of patients was 20.81 ± 5.92 years old. The mean (SD) score of HADS in pregnant, lactating and non-pregnant / non-lactating women were 12.11 (6.72), 11.98 (8.44) and 9.38 (6.2) respectively, and the results showed that the scores in pregnant, lactating women were higher than non-pregnant / non-lactating women (*P* < 0.001). Also the mean (SD) score of QOL and FSFI was 68.29 (9.47), 74.18 (12.65), 79.03 (10.48) and 22.71 (8.16), 22.72 (8.16), 26.19 (3.93) in three groups and the scores in pregnant, lactating women were lower than non-pregnant/non-lactating women (*P* < 0.001).

**Conclusion:**

The COVID-19 epidemic increases the risk of depression, anxiety, FSD, and lowers QoL in pregnant and lactating women, with the general population. This suggests the urgent need for psychological intervention in the maternal population during the epidemic.

## Background

In December 2019, an unknown cause of pneumonia was identified in Wuhan (Hubei, China), and was called the acute respiratory syndrome coronavirus 2 (causing the disease COVID-19) [1]. The COVID-19, as the sixth public health emergency of international concern, has been rapidly spread from its origin throughout the world [2].

Previous studies have shown the widespread and profound impact of outbreaks on people’s mental health that can cause new psychiatric symptoms or aggravate previous mental illness [3]. COVID-19, as a public health crisis, has caused concern and psychological effects on people [4]. A survey of 2,000 people was conducted in the United States during the COVID-19 pandemic, 61% of participants reported being more concerned and anxious about their ability to have children and family planning, and 31% changed their fertility decisions entirely. The two main reasons for delaying fertility during COVID-19 were access to prenatal care and financial reasons [5].

Previous studies on the Severe Acute Respiratory Syndrome (SARS) epidemic have shown that pregnant women are more susceptible to be anxious compared to non-pregnant women; these included anxiety about infection, the transmission of infection to the fetus, acquired infection during childbirth, and teratogenicity of microorganisms and medicines. They were scared of going to hospital and health care centers and postponed their prenatal care [6]. Also, Yanting Wu et al. have argued that reducing physical activity is a modifiable cause of depression during the epidemic [7].

It is highly likely that, birth complications such as lower birth weight, shorter gestational age, vomiting during pregnancy, preeclampsia, lower Apgar scores, and extended hospital stay have been related to perinatal anxiety and depression [8] furthermore they can affect the quality of life (QoL) and sexual function as an important part of it [9, 10].

In order to the high preponderance of the COVID-19 and its adverse psychological effects on people's lives and the lack of studies on the psychological well-being of pregnant and lactating women during the COVID-19 pandemic, it is necessary to assess the impact of COVID-19 on mental health status of pregnant and lactating women. The present study was conducted to determine and compare psychosocial changes associated with COVID-19 and its impact on sexual function and QoL in pregnant and lactating women and compare the results with non-pregnant/non-lactating women in Iran.

## Material and method

The study was conducted as a cross-sectional study. The sample was selected via convenience sampling method and the sample size was estimated 603 using following formula by considering 15% sample loss:

$$\overline{\mu } = 1/k\sum \mu_{j}$$

$${\Delta } = 1/\sigma^{2} \sum \left( {\mu_{j} - \overline{\mu }} \right)^{2}$$

$${\text{N}} = \lambda /\Delta = 17.43/0.1 = 174.3\sim175$$

Confidence coefficient 99% and power 90%, k is number of groups equal to 3. J = 1,2,3, µ1,µ2,µ3 is mean score in three groups, $\overline{\mu }$ and σ is overall mean and standard deviation respectively and λ is equal to 17.43 [11]. Sample size calculation was done using PASS software.

**Fig. 1:**
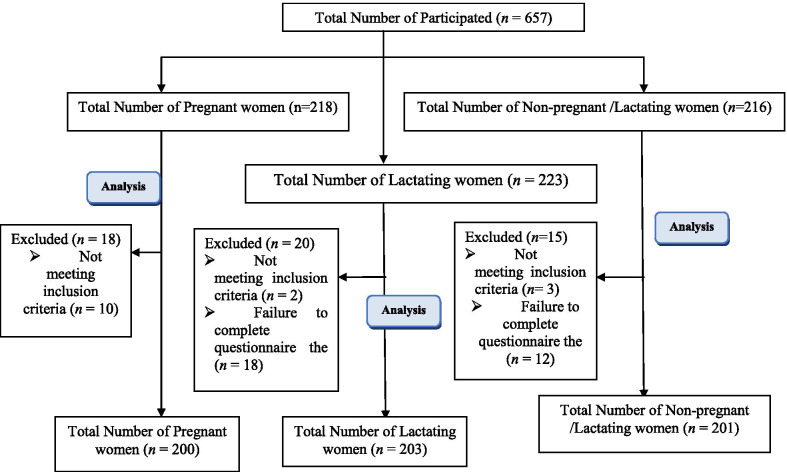
Flow chart for this cross-sectional study

Of the 657 women who completed the questionnaires, 53 women were excluded from the study due to a lack of entry criteria or incomplete filling of questionnaires. Pregnant and lactating women and non-pregnant/non-lactating women within May–June 2020 were included in this study (Fig. 1).

To observe the physical distance to prevent Coronavirus, the web version of the questionnaire which it was the same in terms of questions, words, and order of presentation was accessible for participants through a website. The link of the questionnaires was provided to pregnant women via email or social media by members of the research team. The process of information sorting in the online database was completely automatic and each participant could answer questions with an electronic device just once. Participants' demographic and obstetrical characteristics, depression, anxiety, sexual function, and QoL information was collected through an online questionnaire.

Participants in this study were pregnant and lactating women (infants under one year of age) and non-pregnant/ lactating women between the ages 18 and 45 who could read and write and agreed to complete the online questionnaires, also all of them were exposed to COVISD-19 pandemic. Additionally, they lacked addiction to narcotics and alcohol, were married and living with a husband, and had sexual intercourse in the past four weeks. To avoid potential confounding factors, exclusion criteria included patients under corticosteroid treatment (more than 12.5 mg/dL per week), with a history of chemotherapy, malignancies, organ transplants, HIV, heart disease, high blood pressure, diabetes, underlying respiratory illness, body mass index over 40 and chronic mental illness. The study protocol was approved by the institutional review board and the Ethics Committee of Tarbiat Modares University of Medical Sciences approved the study protocol (IR.MODARES.REC.1399.022).

## Questionnaires

The participants were asked to complete several self-report questionnaires, as follows: they also completed a demographic survey including age, fertility information, duration of menstruation, educational level, occupation, and income.

### Anxiety and depression

The hospital anxiety and depression scale (HADS) using for screening depression and anxiety with 14 questions (7-item for measuring depression and 7-item for anxiety). Higher scores show higher anxiety and depression symptom, a score of 11, and above of it is considered a clinical disease [12]. The validity and reliability of the Iranian version of HADS have been confirmed [13].

### Female sexual function

Female sexual function index (FSFI) is a self-report instrument that measures desire (two items), arousal (four items), lubrication (four items), orgasm (three items), satisfaction (three items) and pain (three items). The total FSFI score has made by the sum of 19 questions scores and overall score 23 was used as the cutoff for clinical female sexual dysfunction (FSD) [14]. The Persian version of FSFI has also been evaluated for both reliability and validity [15].

### Quality of life

Short Form Health Survey (SF-12) questionnaire, a generic instrument for measuring health-related QoL, consisting of 12 items in the physical and mental domains. The total score ranging from 0 to 100, and higher scores indicating a higher health-related QoL [16]. The validity and reliability of this questionnaire have been confirmed in Iran [17].

### Statistics

All statistical analyses were performed by the SPSS software version 22.0 (SPSS Inc., Chicago, IL, USA). Factors associated with QoL were investigated using Spearman and Pearson correlations statistics and multiple regressions. A one-way ANOVA was used [with LSD, Post Hoc test] to compare each variable between groups. Differences were considered significant at *P* < 0.05 for the two tails.

## Result

Table 1 describes the characteristics of each groups. The mean age of participants was 20.81 ± 5.92, the mean duration of marriage was 6.52 ± 4.93 years. There were no significant differences between parity, number of children, education level, and a duration of menstruation (*P* > 0.05).

**Table 1:** Comparison of demographic characteristics between pregnant, lactating and Non-pregnant/lactating groups

Variables	Pregnant group (P) N = 200	Lactating group (L) N = 203	Non-pregnant/non-lactating (N) N = 201	*P* value
Age*	29.69 ± 5.85	30.23 ± 5.18	32.59 ± 6.31	< 0.001
Gravid*	1.92 ± 1.33	1.17.86	1.15 ± 1.10	< 0.001
Parity*	1.05 ± 0.90	1.15 ± 0.82	0.99 ± 0.84	0.21
Number of children*	1.13 ± 0.88	1.16 ± 0.82	1.02 ± 0.83	0.24
Education**				0.41
≤ 12 years	38 (19.2)	30 (14.5)	35 (18.5)	
> 12 years	160 (80.8)	172 (85.5%)	155 (81.5)	
Occupation**				< 0.001
Unemployed	3 (1.6)	2 (1)	5 (2.6)	
Self-employment	144 (75)	151 (75.6)	91 (47.6)	
Student	10 (5.2)	18 (9.1)	26 (13.8)	
Employed	35 (18.2)	29 (14.2)	68 (36)	
Income (Tomans)				< 0.001
≤ 1 Million	137 (72.5)	153 (76.9)	96 (50.8)	
1–3 Million	45 (23.8)	32 (16.4)	56 (29.9)	
> 3 Million	7(3.7)	13 (6.7)	36 (19.3)	
Duration of marriage (year)*	7.03 ± 4.66	7.49 ± 4.61	5.16 ± 5.23	< 0.001
Duration of menstruation*	7.16 ± 1.43	7.24 ± 1.33	7.06 ± 2.88	0.67

^*^Values are given as mean ± SD using *t* test, **values are given as a number (%) using chi-squared test

### Depression and anxiety

There was a statistically significant difference between the mean scores of depression, anxiety, and total HADS in pregnant, lactating and non-pregnant / non-lactating women (*P* <0.001) (Table 2). Although there was no statistically significant difference between lactating with pregnant women in depression, anxiety, and total scores of HADS (*P*  >  0.05).

**Table 2:** Scores for the domain subgroups of HADS between pregnant, lactating and non-pregnant/non-lactating women

Variables	Pregnant group (P) N = 200	Lactating group (L) N = 203	Non-pregnant/non-lactating (N) N = 201	*P* value*		Pair wise comparison *P* value**
**HADS**						
Anxiety	6.59 ± 3.64	7.07 ± 4.91	5.79 ± 3.62	< 0.001	P and N: 0.05 P and L: 0.24 L and N: <0.001	
Depression	5.51 ± 3.75	4.89 ± 4.26	4.04 ± 3.33	< 0.001	P and N: > 0.001 P and L: 0.10 L and N: 0.02	
Total scores	12.11 ± 6.72	11.96 ± 8.44	9.83 ± 6.28	< 0.001	P and N: <0.001 P and L: 0.83 L and N: <0.001	

HADS: Hospital Anxiety and Depression Scale

* One-way ANOVA

**One-way ANOVA followed by LSD Post Hoc test

### Sexual function status

Evaluation of FSFI scores showed that all mean values were higher in non-pregnant / non-lactating women. There was a statistically significant difference between lactating and pregnant women and non-pregnant / non-lactating women in terms of desire, arousal, orgasm, pain, and total FSFI scores (*P *< 0.05) (Table 3).

**Table 3:** Comparison of FSFI and its domains between pregnant, lactating and Non-pregnant/ noon-lactating groups

Variables	Pregnant group (P) N = 200	Lactating group (L) N = 203	Non-pregnant/ non-lactating (N) N = 201	*P* value*	Pair wise comparison *P* value**
Desire	3.25 ± 1.02	3.16 ± 1.06	3.66 ± 0.35	<0.001	P and N: <0.001 P and L: 0.30 N and L: <0.001
Arousal	3.62 ± 1.63	3.62 ± 1.70	4.53 ± 1.06	<0.001	P and N: <0.001 P and L: 0.97 N and L: <0.001
Lubrication	3.89 ± 1.79	3.91 ± 1.71	4.09 ± 1.17	0.36	P and N: 0.19 P and L: 0.87 N and L: 0.25
Orgasm	3.75 ± 1.87	3.91 ± 1.81	4.35 ± 1.01	<0.001	P and N: <0.001 P and L: 0.32 N and L: 0.007
Satisfaction	4.37 ± 1.42	4.25 ± 1.46	4.50 ± 1.13	0.18	P and N: 0.35 P and L: 0.36 N and L: 0.06
Pain	3.81 ± 1.97	3.79 ± 1.88	5.03 ± 1.07	<0.001	P and N: <0.001 P and L: 0.92 N and L: <0.001
Total score	22.71 ± 8.16	22.72 ± 8.16	26.19 ± 3.93	<0.001	P and N: <0.001 P and L: 0.92 N and L: <0.001

FSFI: Female Sexual Function Index, TL: tubal ligation. ANOVA: analysis of variance

^*^ One-way ANOVA

^**^ One-way ANOVA followed by LSD Post Hoc test

According to the Table3, there was a significant difference between the mean score of lubrication in between groups (*P* < 0.05). There was no significant difference between the scores of the pregnant and lactating women in all sub-groups of sexual function (*P* > 0.05).

Lactating and pregnant women experienced more FSD than non-pregnant / non-lactating women (P < 0.001). About 37% of the pregnant and lactating women and 54% of the non-pregnant / non-lactating women, reporte a dysfunction in the lubrication dimension (P < 0.001) (data not shown).

### Quality of life status

Based on the Table 4, mean total scores of SF-12 in the pregnant, lactating, and non-pregnant / non-lactating women were 68.29 ± 9.47, 74.18 ± 12.65, 79.03 ± 10.48, respectively (*P* < 0.001). As Table 4 shows, the pregnant women reported significantly less physical, psychological, and total scores of QoL than the other two groups (*P* < 0.001). There was no significant difference between pregnant and lactating women in mental and total scores of QoL (*P* > 0.05).

**Table 4:** Scores for the domain subgroups of QoL, between pregnant, lactating and non-pregnant / non-lactating women

Variables	Pregnant group (P) N = 200	Lactating group (L) N = 203	Non-pregnant/ non-lactating (N) N = 201	*P* value*	Pair wise comparison *P* value**
Sum score physical Components (PCS-12)	69.95 ± 12.62	79.42 ± 13.61	84.45 ± 11.11	<0.001	P and N: <0.001 P and L: <0.001 L and N: <0.001
Sum score mental Components (MCS-12)	67.31 ± 13.53	68.93 ± 14.72	73.61 ± 13.64	<0.001	P and N: << 0.001 P and L: 0.24 L and N: <<0.001
Total score	68.29 ± 9.47	74.18 ± 12.65	79.03 ± 10.48	<0.001	P and N: <0.001 P and L: 0.83 L and N: <0.001

QoL: Quality of Life, SF-12: Short Form-12

^*^ One-way ANOVA

^**^ One-way ANOVA followed by LSD Post Hoc test

## Discussion

The number of patients and suspicious are increasing and the uncertainty and low predictability of pandemic threaten people’s mental health [18]. To our knowledge, our study was among one of the first studies to investigate the impact of the COVID-19 pandemic on the depression, anxiety, sexual function, and QoL of pregnant and lactating women and compare the results with non-pregnant /lactating women in Iran.

### Depression and anxiety

In the results of this study, pregnant and lactating women got significantly lower scores in both dimensions of mental status (depression and anxiety), compared to the non-pregnant / non-lactating women.

These results seem to be consistent with Wu et al. research, which found the impact of COVID-19 awareness on the increasing prevalence of prenatal depression (PND). The trend of PND prevalence increased with the number of death and newly-diagnosed patients, lack of access to the features of the disease, fear of infection, and vertical transmission from mother to fetus [19].

Due to the high prevalence of Covid-19, the WHO has proposed quarantine to reduce human-to-human transmission [20]. Many symptoms of mental distress such as depression, stress, irritability, and insomnia in those who had been quarantined, have been reported higher. Quarantine stressors include longer duration of quarantine, fear of infection, loss of normal life routine, reduced social activity, and physical contact with others, having inadequate basic supplies, lack of sufficient information, and clear guidelines on actions to take, and serious socioeconomic problems [21].

Previous epidemic (SARS) experiments have shown that pregnant women suffer from high levels of anxiety, especially those who are more emotionally vulnerable [22].

Stress and anxiety suppress the immune system and make people susceptible to infectious diseases [23] and various studies have reported an association between mental morbidity during pregnancy and adverse outcomes of pregnancy such as low birth weight and preterm labor SPS:refid::bib^24^(24). In addition, pregnancy and postpartum depression are both associated with shorter breastfeeding duration, likewise, mother's anxiety is associated with breastfeeding difficulties, shorter breastfeeding intention, and duration of breastfeeding [25]. The cumulative effect of the mental burden imposed on society by COVID-19 along with pregnancy and lactating as a mentally sensitive period may be a possible explanation for these results.

Given the devastating effects of anxiety and depression on the immune system, pregnancy, and lactating, these results emphasize the importance of mental health care for pregnant and lactating mothers in outbreak duration.

### Sexual function status

Sexual function as a physical, emotional, and mental state is an essential part of each human being’s personality and the cornerstone of a couple's relationship; it also has a significant impact on QoL [26]. The vast majority of studies showed that sexual function decreases significantly during pregnancy, and this decline can be continued for the first 3–6 months after delivery [27]. The present study also demonstrated that there was a significant FSD in pregnant and lactating women.

Yuksel et al. [28] compared the frequency of sexual intercourse, desire for pregnancy, and FSFI scores among women during the COVID-19 pandemic with 6–12 months prior to the pandemic. They reported higher sexual desire and frequency of sexual intercourse whereas the lower quality of sexual life during the COVID-19 pandemic. The study also found a significant reduction in the number of women planning to become pregnant, which could result in fears about its possible effects on the fetus, difficult access to the health system, and economic problems. However, high levels of chronic stress in other disasters have led to a decrease in sexual desire and intercourse [29].

Although not much data is available, unemployment due to quarantine, anxiety about job security, worry about personal and family health, and the ability to have access to medical care can affect sexual desire howsoever some people may resort to sex for comfort or a temporary distraction [30]. Anxiety disorder and depression symptoms caused by “hypochondriac concerns” (worry about being infected) [31] and the proven effect of anxiety and depression on sexual function [32] may explain increased sexual dysfunction in COVID-19 outbreak.

The results of this study did not show a significant difference in FSD between pregnant and lactating women. Both pregnancy and lactation can affect sexual function through physical changes (including fatigue, back pain, dyspareunia, urinary tract infections, and vaginitis), hormonal changes (changed levels of estrogen, progesterone, and prolactin), and psychogenic factors (such as the anxiety of delivery and motherhood, relationship, lack of self-esteem, sexual guilt, and specific concerns about body image and general health status) [33].

According to low estrogen and progesterone levels and high levels of prolactin during lactating [34] and increasing blood vessels in the vagina and decreased sexual arousal in pregnancy that can lead to dryness[35]; contrary to our expectations, this study found more prevalence of lubrication dysfunction in non-pregnant /lactating women.

Given to considerable impacts of pregnancy [36] and lactation [37] on sexual activity by the many significant physical and mental changes, pregnant and lactating women are more prone to the effectiveness of the mental impact of COVID-19 and sexual dysfunction.

### Quality of life status

QoL is defined as people's perception of their position in life within their cultural and value contexts, which relates to their goals, expectations, standards, and concerns [38].

In terms of the factors associated with QoL, an increased rate of depression, anxiety, sleep disorders, and experience of the life-threatening events were associated with a poor QoL during pregnancy [39]. The results of this study, therefore, indicate significantly lower mental components of QoL during the pandemic in pregnant and lactating women (P<0.001). Possible reasons are the economic effects of quarantine and epidemic, the unpredictable future, and the fear of the infant's health. Our study results were consistent with the findings reported by Lau et al., who investigated mental health and QoL in Hong Kong residents during the SARS epidemic [40].

Han Xiao reported the effect of anxiety and stress of COVID-19 quarantine on sleep quality such as difficulty falling asleep, or wake up easily [41] also, Shao-YuTsai demonstrated a high prevalence of sleep disturbances in pregnant women [42]. There are several possible explanations for lower QoL in pregnant women during COVID-19; one of them is the cumulative effects of these factors and the importance of quality of sleep in QoL.

The results of this study indicated a lower score of physical QoL throughout pregnancy than lactating and non-pregnant / non-lactating women, particularly related to decreased physical activity and physical symptoms such as nausea and vomiting, epigastralgia, reflux, shortness of breath, dizziness, back pain, and sleep problems [43].

One of the limitations of this study was the lack of accurate information about the mental and sexual profile of participants before the pandemic. Another one was that the questionnaires were filled online and if participants needed additional information about the questions, no professional was available.

Based on our knowledge, there are reasonably good understanding of the correlation between pregnancy, postpartum period and depression, but there is almost nothing about the relationship between COVID-19 pandemic and mental health, sexual function, and QoL. Since the COVID-19 pandemic is still ongoing, these findings need to be confirmed and investigated in future larger population studies.

## Conclusions

To sum up, it can be said that pregnant women and mothers in the post-partum period as high-risk groups for mental disorder and sexual dysfunction, are more susceptible to the possible psychological effects of coronavirus pandemic. therefore, it seems that pay more attention to the mental health care services as a boon to combat the psychological effects of coronaviruses leads to reduce the consequences of depression, anxiety, and stress for the unborn/newborn child.

## Data Availability

The data sets used and analyzed during the current study are available from the corresponding author on reasonable request.

## Abbreviations

FSFI: Female Sexual Function Index
QOL: Quality of life
SF-12: Short Form Health Survey
SARS: Severe Acute Respiratory Syndrome
COVID19: Coronavirus disease 2019
WHO: World Health Organization
HADS: Hospital Anxiety and Depression Scale
